# Exploring the Cognitive Model of Social Anxiety in Autistic Young People—The Central Role of Bodily Symptoms

**DOI:** 10.1007/s10803-021-05359-0

**Published:** 2021-12-04

**Authors:** H. Wood, S. Rusbridge, J. Lei, C. Lomax, J. Elliston, A. Russell

**Affiliations:** 1 Child and Adolescent Mental Health Service, Somerset NHS Foundation Trust, Taunton, TA2 7PG England; 2 Child and Adolescent Mental Health Service, NHS Lothian, Edinburgh, EH26 8EZ Scotland; 3 Department of Psychology, Institute of Psychiatry, Psychology and Neuroscience, Kings College London, London, SE5 8AF England; 4 School of Psychology, Newcastle University, Newcastle upon Tyne, NE1 7RU England; 5 Oxford Institute of Clinical Psychology Training and Research, Oxford, OX3 7JX England; 6 Centre for Applied Autism Research, Department of Psychology, University of Bath, Bath, BA2 7AY England

**Keywords:** Autism, Social anxiety, Cognitive behaviour theory

## Abstract

We explored the role of negative performance beliefs and self-focused attention considered central to psychological models of social anxiety but not studied in autism. Firstly, we compared self- and observer ratings of performance on a social task for 71 young autistic people, 41 high and 30 low in social anxiety, finding a significant main effect of social anxiety but not rater. Subsequently, 76 autistic young people, 46 high and 30 low social anxiety completed measures of interoceptive sensibility and focus of attention following a social task. Only heightened interoceptive sensibility fully mediated the relationship between self-ratings of social performance and social anxiety. These findings suggest awareness of bodily sensations are critical to anxiety in social situations with implications for treatment.

Social anxiety disorder (SAD) is one of the most common anxiety disorders in adolescents (Jefferies & Ungar, 2020), and is defined by an intense anxiety or fear about entering social situations where the individual fears negative evaluation from others and consequently avoids social situations (American Psychiatric Association, 2013). A recent study that explored SAD in 6825 neurotypical young people across seven different countries estimated a prevalence rate of 36% for those who met the threshold for SAD diagnosis, with an additional 18% who did not perceive themselves to have SAD but still surpassed the diagnostic threshold (Jefferies & Ungar, 2020). The prevalence rate of SAD is also estimated to be between 29.2% and 57% for autistic^1^ children and adolescents, (Bellini, 2004; Kuusikko et al., 2008; Simonoff et al., 2008) with population ascertainment and measurement methods relevant to the differing ranges reported (see Spain et al., 2018 for a review). There is some debate as to whether the high co-occurrence rate between the two conditions reflects a “true” comorbidity (Mason & Scior, 2004; Wood & Gadow, 2010) given that both may affect social communication e.g. avoidance of social situations may derive from fear of negative evaluation as characterised by SAD, or may reflect individual preference on the basis of autism-related social communication differences. Differences in reporting internal states and/or reduced verbal ability in autism may contribute to all clinically relevant phenomena being attributed to a neurodevelopmental diagnosis. The involvement of verbally able autistic adolescents and adults has furthered understanding in this area significantly. Research that examined construct overlap across self-report measures of social anxiety and autistic traits in neurotypical and autistic adolescents have found that social anxiety measures specifically assessed cognitive processes during social interaction and performance (White et al., 2012) such as fear of negative evaluation (Lei & Russell, 2020), while autistic traits captured a broader range of social and non-social difficulties ranging from theory of mind to preference for routine (Lei & Russell, 2020; White et al., 2012). Cognitive processes related to social anxiety may also influence an individual’s beliefs about their own social competence, which may be more relevant to social anxiety rather than objective social skills, as studies found that neurotypical adolescents with high social anxiety rated their performance more negatively than observers, whereas no such discrepancies were found for adolescents with low social anxiety (Cartwright‐Hatton et al., 2003, 2005).

## Cognitive Model of Social Anxiety

The cognitive model of social anxiety focuses on the role of distorted beliefs (Clark & Wells, 1995), and has become the dominant treatment model in neurotypical individuals with good treatment outcomes (National Institute for Health and Care Excellence, 2013) and an empirically-grounded evidence-base (Clark, 2001). The model postulates that individuals with social anxiety develop negative self-beliefs, high standards for social performance and catastrophic beliefs about failure. On entering a social situation, the focus of attention shifts from others to the self to monitor social performance, and self-focused attention (SFA) is defined as “an awareness of self-referent, internally generated information that stands in contrast to an awareness of externally generated information derived through sensory receptors” (Ingram, 1990, p. 156). Internal information obtained via SFA is used to create an image of how oneself is perceived by others, often disproportionately negative and based on emotions rather than reality (e.g., ‘I *feel* anxious therefore I must *look* anxious’). SFA reduces attendance to positive feedback from others (Clark, 2005; Hope et al., 1990; Pozo et al., 2016), preventing disconfirmation of negative self-beliefs. Self-focused attentional processes in-situ and trait SFA have been found to have a strong relationship with social anxiety (see Norton & Abbott, 2016 for a review).

There is evidence supporting the role of SFA in maintaining negative performance beliefs in neurotypical individuals, such that those with greater social anxiety reported higher levels of SFA and physiological sensations than those with lower social anxiety during a social situation (Mellings & Alden, 2000). The ability to detect and consciously perceive one’s physiological sensations and relate to internal body experiences is known as interoception (Craig, 2003). Interoception is a multidimensional construct that includes objective measures of performance accuracy on behavioural measures for example tracking one’s heart rate (interoceptive accuracy), subjective perception and experience of one’s bodily sensations (interoceptive sensibility) and a metacognitive reflection on one’s awareness of their ability to accurately perceive internal processes (interoceptive awareness) (Garfinkel et al., 2015, 2016). For non-autistic individuals with high levels of social anxiety, interoceptive sensibility was positively correlated with overestimating negative aspects of social performance (Mansell & Clark, 1999). Later studies also found that individuals with high social anxiety who held in mind an observer-perspective image of themselves had greater interoceptive sensibility and perceived their performance more negatively than individuals with low social anxiety (Makkar & Grisham, 2011; Vassilopoulos, 2005). Studies have found that measures of physiological sensations relevant to anxiety including heart rate, respiratory rate, blood pressure and skin conductance do not *objectively* differ between individuals with high versus low social anxiety during social performance tasks such as giving a speech or unstructured social interaction (Anderson & Hope, 2009; Mauss et al., 2004), and therefore SFA may increase the subjective perception or interoceptive sensibility of anxiety-related physiological sensations, which contribute to developing overly negative beliefs about social performance (Domschke et al., 2010).

## Autism, Interoceptive Sensibility and Self-focused Attention

Whilst the cognitive model of social anxiety has a well-established evidence-base for neurotypical individuals (Clark & Wells, 1995), its applicability for autistic adolescents is less clear. Differences in interoceptive sensibility has been found in autistic adults compared to neurotypical adults, such that autistic adults show enhanced interoceptive sensibility but reduced interoceptive accuracy when asked to track their own heartbeat (Garfinkel et al., 2015, 2016), and this discrepancy between interoceptive sensibility and accuracy, known as interoceptive trait prediction error (ITPE), significantly predicted anxiety above and beyond the effects of autism severity (Garfinkel et al., 2016). The relationship between ITPE and anxiety has also been replicated in autistic children and young people aged 6–18 years old compared to their neurotypical peers (Palser et al., 2018). Specifically, in a sample of autistic and neurotypical adolescents aged 11–18 years old, Pickard et al. (2020) found that it was interoceptive sensibility, and not interoceptive awareness which was associated with greater social anxiety in both groups. However, none of the studies examined whether heightened interoceptive sensibility amongst autistic adolescents was uniform across different body areas for those who experience high versus low levels of social anxiety, to explore whether there may be specific physiological markers related to social anxiety per se.

The potential hypersensitivity to one’s physiological arousal in autism is consistent with the developmental pathway model of social anxiety in autism, which suggests that autistic children may experience greater physiological arousal that can be difficult to regulate and are consequently vulnerable to developing social anxiety following negative social encounters (Bellini, 2004). Difficulties coping with physiological arousal can lead to withdrawal, reducing opportunities for social skills development and maintain social anxiety and avoidance (Bellini, 2004). However, a recent literature review exploring the empirical evidence base of differences in physiological arousal in autism have found very mixed evidence with limited scope to draw a conclusion that is fully consistent with increased physiological arousal in this population (Arora et al., 2021). One study found that there is evidence for a positive correlation between cortisol response and age amongst autistic young people over the course of adolescence independent of pubertal stage, who also show a blunted stress response when compared to their neurotypical peers (Corbett et al., 2021), which led the authors to hypothesise that it is the time lag between the development of threat appraisal and stress response that interferes with the ability to self-regulate in social situations for older autistic adolescents. Despite there being some evidence to suggest that interoceptive sensibility may play a role in social anxiety in autism, no studies to date have directly compared whether autistic adolescents may also have more negative beliefs about their social competence compared to objective observations of their social skills, and to what extent the relationship between self-focused attention and awareness of bodily sensations in social situations may relate to social anxiety.

## Current Study

The current study aimed to explore the relevance to autism of two components of the cognitive model of social anxiety as proposed by Clark and Wells (1995) across two studies. Firstly, we investigated the role of negative performance beliefs by asking whether autistic young people with high levels of social anxiety report a more negative perception of their social performance compared to observer ratings following participation in a group discussion task. Secondly, we asked whether negative self-ratings of social performance are related to the level of anxiety experienced in a social situation and whether interoceptive sensibility and/or more general SFA processes mediate this relationship. We also explored patterns of interoceptive sensibility across different body areas for those with high versus low levels of social anxiety.

## Methods

### Participants

Participants were recruited through specialised educational provisions, universities, charities and social groups specifically for autistic young people in the region of [LOCATION ANONNYMISED FOR REVIEW]. All participants had a confirmed diagnosis of ASD in order to access specialised educational provision and otherwise provided confirmation of ASD diagnosis by sharing clinical letter/report with the research team. Participants were recruited separately for two studies with a shared focus of understanding the application of cognitive models of social anxiety in autistic young people and data was combined for the current analysis. Inclusion criteria for participation in both studies were similar: (1) aged between 13 and 25 years (inclusive); (2) English-speaking; (3) previous diagnosis of ASD. Exclusion criteria were: (1) diagnosed intellectual disability; (2) substance misuse; (3) a physical health or neurological condition affecting the central nervous system which may result in differences in interoceptive processing or sense of the felt self. The rationale for the defined age range is related to the maturation of the adolescent brain, which begins in early adolescence reaching a peak in development by the age of 25 years (Arain et al., 2013) with findings from Diffusion Tensor Imaging studies of white matter tract values underpinning this (see Lebel & Deoni, 2018 for a review) peak between ages 21 and 25 years suggesting brain maturation from a structural connectivity perspective with reduced inter-individual variability. A total of 87 autistic young people between the ages of 14 and 21 years participated in the first part of the study. A total of 76 autistic young people between the ages of 16 and 25 years took part in the second part of the study.

### Measures

Detailed descriptions for each of the measures used in the current study, their modifications, and internal consistency can be found in Appendix 1. In brief, social anxiety was measured by using Social Anxiety Scale for Adolescents (SAS-A, La Greca & Lopez, 1998), and Social Phobia Inventory (SPIN, Connor et al., 2000). A modified version of the Focus of Attention Questionnaire (FAQ) as described in Mellings and Alden (2000) was used to assess SFA during the social situation, and Self-Consciousness Scale (SCS, Fenigstein et al., 1975) was used to assess trait SFA. A modified version of the specific form of the Autonomic Perception Questionnaire (APQ, Mandler et al., 1958) was used to assess interoceptive sensibility during the social situation. Modifications included the use of visual cues to depict the bodily domain being enquired about for groups of items and amendment of terminology by replacing less frequently used terms with those used more frequently e.g., replacing ‘perspiration’ with ‘sweat’. A modified version of the Performance Scale (Cartwright-Hatton et al., 2005; Cartwright‐Hatton et al., 2003) was used to assess self- and observer-ratings of social performance during the social situation. Items were modified to reflect the use of a filmed group discussion task in contrast to the individual speech in the Cartwright-Hatton et al. (2005) study and reduce the use of metaphor in one item i.e. amending ‘stumble over your words’ to ‘speaking confidently’. Items were presented as statements with which participants and observers could rate agreement on a 4-point likert scale ranging from ‘1’—strongly disagree, ‘2’—disagree, ‘3’—agree and ‘4’—strongly agree’ with 2 items (‘I appeared nervous’ and ‘I blushed’) reverse scored.

### Procedure

The study was approved by the [INSTITUTION NAME ANNONYMISED FOR REVIEW] Ethics Committee. Approval was also gained from the relevant county council in the UK. Participants were given a written project information sheet with a separate version for parents. Parental consent was obtained for all participants under the age of 18 years. All participants gave written informed consent prior to taking part.

The study was carried out in the education or social group setting. Participants in both studies completed questionnaires on anxiety (SAS-A) prior to the group task, and participants in study part two also completed a measure of self-consciousness (SCS). Participants then took part in the social performance task. This was a group discussion following the showing of a short piece of film available from *YouTube* entitled ‘*Channel 4 Paralympics—Meet the Superhumans’* (Meet the Superhumans, 2012). This critically acclaimed film was selected as it was deemed to have content that was neutral enough to appeal to a wide audience as opposed to being related to any particular interests. Groups consisted of between three to five participants and one researcher. A research assistant was also present for groups of fewer than three participants. The research assistant had some involvement in the discussion and was present to increase the social demand by increasing the number of people present. During the discussions the researchers were blind to participants’ response on the measures.

After watching the short film, participants were asked what they thought about the film, if they thought it was effective and why. The conversation was free to follow any relevant areas of discussion. Each person was invited to contribute to this discussion, however there was no pressure to do so if they did not want to speak. Discussion lasted approximated 10–15 min and was filmed using a digital camcorder. Participants were informed a priori that the discussion would be recorded and subsequently viewed by an observer.

After taking part in the group discussion, participants in both studies were asked to complete the performance ratings, and participants in study part two also completed measures of anxiety (SPIN), interoceptive sensibility (APQ) and focus of attention (FAQ). Two researchers later watched the footage and completed the observer scale of the performance questionnaire, blind to the participants’ questionnaire scores and self-performance ratings. Observer ratings were discussed, and a consensus rating was reached. A randomly selected sample (15%) of all participants were independently rated by a research assistant (JE). The intra-class correlation coefficient using a 2-way mixed model with absolute agreement was 0.882 (95% Confidence Interval 0.600–0.966).

### Analysis

Data were analysed using IBM SPSS Version 25 (Hill, 2017). Study part one examined whether social anxiety affected the discrepancy between self- and observer-performance ratings amongst autistic young people. Participants were grouped into high (≥ 50) and low (< 50) social anxiety according to their self-reported ratings of social anxiety based on the recommended cut-off score of 50 on the SAS-A (La Greca, 1999). Group differences in anxiety and age were assessed using independent samples t-test, and gender difference was examined using chi-squared test. These data were gathered from participants recruited to each study in 3 phase or streams. In order to consider any cohort effects, a one-way ANOVA was used to investigate differences in outcome measures across three recruitment groups for each part of the study, with Bonferroni to correct for multiples comparisons. A two-way ANOVA was used to investigate the hypothesised interaction between social anxiety level (high/low) and performance rating (self/observer).

Study part two explored the extent to which interoceptive sensibility (APQ), state SFA (FAQ-Self subscale), and trait SFA (SCS-Public subscale) mediated the relationship between self-performance ratings and social anxiety (SPIN) through three simple mediation models. For this second part of the study, a one-way ANOVA was used to investigate differences in outcome measures across three recruitment groups, with Bonferroni to correct for multiple comparisons. We then conducted Pearson’s correlation between self-performance rating and social anxiety, as well as correlations between state and trait SFA and interoceptive sensibility. Next, we conducted simple mediation analyses using PROCESS Macro Version 3 (Hayes, 2017) by following the steps outlined by Kane and Ashbaugh (2017). We first checked to ensure that the predictors within each model met assumptions of linearity, homoscedasticity, normality of estimation error and independence of observations. Next, we ran mediation models using bias-corrected bootstrapping method with 5000 samples. Finally, we also conducted independent samples t-test and chi-square test of independence to explore whether there are differences in the level of interoceptive sensibility scores and endorsement for each of the eight body areas noted by the APQ for participants with high versus low levels of social anxiety (i.e., scored above and below the SPIN cut-off scores).

## Results

### Self and Observer Performance Rating Discrepancy

Demographic information and group differences for participants in study part one is shown in Table 1. Across the three main groups of participants recruited, we did not find any between group differences on the social anxiety scale total score [*F* (2, 68) = 0.804, p = 0.452], nor any of the subscales including fear of negative evaluation [*F* (2, 68) = 0.340, p = 0.713], social avoidance and distress in general [*F* (2, 68) = 1.375, p = 0.259], nor new situations [*F* (2, 68) = 1.416, p = 0.250]. There were also no differences between groups on self-performance rating [*F* (2, 68) = 0.687, p = 0.506]. There were no differences in the relative proportion of male or female young people in the high social anxiety group (n = 41) compared to the low social anxiety group (n = 30, p = 0.112), and both groups were matched on age. The high social anxiety group reported greater fear of negative evaluation by others, greater social anxiety in general and in new situations, as well as greater overall levels of social anxiety compared to the low social anxiety group (p < 0.001 for all). Results from the two-way ANOVA showed a significant main effect of level of social anxiety [*F*(1, 69) = 4.70, partial ε^2^ = 0.064, p = 0.034], as those who experienced higher levels of social anxiety received poorer performance ratings compared to those who experienced lower levels of social anxiety. There was no significant main effect of rater [*F*(1, 69) = 0.605, partial ε^2^ = 0.009; p = 0.439], and no significant rater by anxiety interaction [*F*(1, 69) = 0.239, partial ε^2^ = 0.003, p = 0.626], suggesting that the discrepancy between performance ratings for young people with high versus low levels of social anxiety did not differ based on whether the rating was made by the observer or the young person themself.

**Table 1:** Study one: demographic information for participants and group differences between those with high versus low social anxiety (n = 71)

	High social anxiety (n = 41)	Low social anxiety (n = 30)	Group differences (High vs. Low)
Gender	n (%)		*Χ*^*2*^ (*p*)
Male	29 (70.73)	26 (86.67)	2.52 (.112)
Female	12 (29.27)	4 (13.13)	
	M (SD)		*t* (85) (*p*)
Age (Years)	18.17 (2.46)	17.33 (1.42)	1.81 (.075)
SAS-A			
FNE	26.90 (5.64)	16.10 (5.37)	8.13 (< .001)
General	13.46 (2.53)	8.10 (2.78)	8.46 (< .001)
New situations	22.36 (3.18)	15.03 (4.10)	8.50 (< .001)
Total	62.73 (7.30)	39.13 (8.60)	12.48 (< .001)
Performance Rating			
Self	24.51 (4.62)	27.03 (3.98)	− 2.41 (.019)
Observer	25.39 (5.70)	27.23 (5.76)	− 1.34 (.184)

*SAS-A* social anxiety Scale—adolescents, *FNE* fear of negative evaluation

### Factors Underlying Self-performance Rating and Social Anxiety

Demographic information for participants in part two of the study is shown in Table 2. Across the three recruitment groups, no significant between group difference were found across any of the outcome measures, including social anxiety as measured by SAS-A [*F* (2, 72 = 2.469, p = 0.092] and SPIN [*F* (2, 73) = 1.807, p = 0.171], self-consciousness scale {private [*F* (2, 73) = 0.957, p = 0.389]; public [*F* (2, 73) = 1.823, p = 0.169]; social anxiety [*F* (2, 73) = 0.384, p = 0.683]}, autonomic perception questionnaire total [*F* (2, 73) = 0.186, p = 0.831], and focus of attention {self [*F* (2, 73) = 1.188, p = 0.311]; other [*F* (2, 73) = 0.646, p = 0.527]}. Due to lack of between group differences, group was not used as a covariate for the analyses below.

**Table 2:** Study two: demographic information for participants (n = 76)

	M (SD)	Range
Gender	(n)	(%)
Male	44	57.89
Female	32	42.11
Age (Years)	17.91 (1.93)	16–25
SAS-A (n = 75)		
FNE	22.28 (6.74)	10–38
General	10.72 (3.83)	4–22
New situations	18.88 (5.08)	6–29
Total	51.84 (12.89)	20–82
SPIN		
Fear	8.90 (4.90)	0–20
Avoidance	10.49 (5.88)	0–26
Physical	4.50 (4.11)	0–17
Total	23.88 (13.44)	0–58
Self—performance rating	25.13 (4.01)	15–35
SCS		
Private	22.28 (5.29)	12–34
Public	16.26 (5.29)	3–27
Social anxiety	13.09 (5.31)	0–24
FAQ		
Self	1.93 (0.66)	1–4
Other	2.25 (0.64)	1.1–3.8
APQ	36.88 (29.35)	0.2–133.3
General awareness	8.74 (6.22)	0–25.4
Blood pressure	1.12 (2.12)	0–8.3
Temperature	4.35 (4.48)	0–17.6
Perspiration	1.25 (2.04)	0–9
Muscle tension	7.23 (7.07)	0–36.7
Heart rate	5.16 (6.28)	0–20.1
Respiration	4.50 (5.35)	0–19.3
Gastrointestinal	4.76 (5.64)	0–30.3

*SAS-A* Social anxiety scale for adolescents, *SPIN* Social phobia inventory, *SCS* social consciousness scale, *FAQ* focus of attention questionnaire, *APQ* autonomic perception questionnaire

Pearson’s correlation showed that participants with poorer self-rated performance also had higher levels of social anxiety as measured by SPIN (*r* = − 0.415, p < 0.001). Higher social anxiety was also associated with higher interoceptive sensibility as measured by APQ (*r* = 0.612, p < 0.001), state SFA as measured by self-subscale of FAQ (*r* = 0.583, p < 0.001), and trait SFA as measured by public-subscale of SCS (*r* = 0.332, p = 0.003). Higher interoceptive sensibility was also significantly associated with both higher state SFA (*r* = 0.778, p < 0.001) and trait SFA (*r* = 0.345, p = 0.002).

**Fig. 1:**
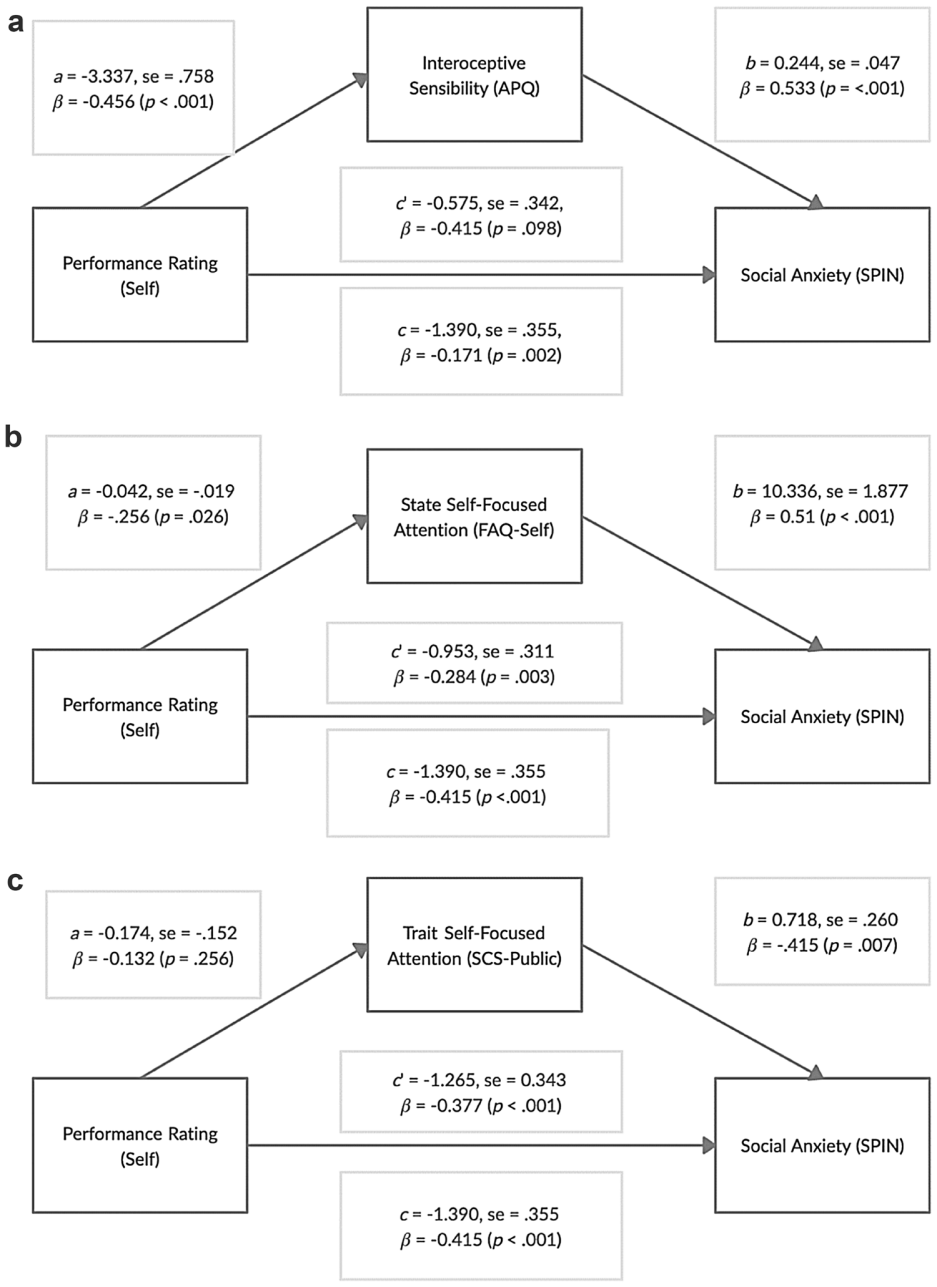
Simple mediation models assessing **a** interoceptive sensibility, **b** state self-focused attention, **c** trait self-focused attention as potential mediators between self-performance rating and social anxiety. *APQ* autonomic perception questionnaire, *SPIN* social phobia inventory, *FAQ* focus of attention questionnaire, *SCS* social consciousness questionnaire, *c* total effect, *c’* direct effect. Unstandardised coefficients and their standard errors, and standardised coefficients are reported

Results from all three simple mediation analyses are shown in Fig. 1. The first simple mediation analysis indicated that self-performance rating is indirectly related to social anxiety through its relationship with interoceptive sensibility (Fig. 1a). Those with lower self-performance ratings had greater interoceptive sensibility (a = − 3.337, p < 0.001), and greater interoceptive sensibility was subsequently related to greater social anxiety (b = 0.244, p < 0.001). The indirect effect of self-performance rating via interoceptive sensibility was negative (ab = − 0.816), and 95% bias-corrected confidence interval based on 5000 bootstrap samples was entirely below zero (− 1.427, − 0.334). Lower self-performance rating was no longer associated with greater social anxiety after taking into account the indirect effect through interoceptive sensibility (c’ = − 0.575, p = 0.098).

Results from the second simple mediation analysis indicated that self-performance rating is not related to social anxiety through its relationship with SFA (Fig. 1b). Lower self-performance rating was still associated with greater social anxiety after taking into account the indirect effect through state SFA (c’ = − 1.390, p < 0.001). The indirect effect of self-performance rating via state SFA was negative (ab = − 0.438), and 95% bias-corrected confidence interval based on 5000 bootstrap samples ranged between − 0.905 and 0.002. Results from the third simple mediation analysis indicated that self-performance rating is not related to social anxiety through its relationship with trait SFA (public self-consciousness) (Fig. 1c). Lower self-performance rating was still associated with greater social anxiety after taking into account the indirect effect through public social consciousness (c’ = − 1.265, p < 0.001). The indirect effect of self-performance rating via trait SFA was (ab = − 0.124), and 95% bias-corrected confidence interval based on 5000 bootstrap samples ranged between − 0.486 and 0.089.

Table 3 shows a comparison of the breakdown of level of endorsement for different bodily areas for participants with high (n = 46) versus low (n = 30) levels of social anxiety (based on scoring above or below the cut-off score on SPIN). Although a chi-square test of independence showed that the proportion of participants who endorsed sensations in each of the eight bodily areas did not differ by level of self-reported social anxiety, independent sample t-tests showed that those who had higher levels of social anxiety reported significant greater interoceptive sensibility in bodily temperature, muscle tension and heart rate.

**Table 3:** Study two: differences in interoceptive sensibility (total scores and number of participants who endorsed at least one item for each bodily area) as measured by autonomic perception questionnaire (APQ) across participants who scored above and below the cut-off score on the social phobia inventory

Physiological domain		Total scores				Endorsing ≥ 1 item above 0%, n (%)				
Domain	Items	M (SD)		*t* (74) (High vs. Low)	*p* value	n (%)			*Χ*^*2*^	*p* value
		Low (n = 30)	High (n = 46)			Total (n = 76)	Low (n = 30)	High (n = 46)		
General awareness	3	7.37 (4.92)	9.63 (6.85)	1.56	.124	73 (96.05)	29 (96.67)	44 (95.65)	0.05	.824
Blood pressure	1	0.64 (1.62)	1.43 (2.36)	1.60	.115	55 (72.37)	9 (30)	20 (43.48)	1.40	.237
Temperature	3	2.44 (2.68)	5.59 (4.99)	3.17	.002*	57 (75)	19 (63.33)	38 (62.61)	3.60	.058
Perspiration	1	0.66 (1.30)	1.63 (2.33)	2.08	.041	41 (53.95)	11 (36.67)	30 (65.22)	5.96	.015
Muscle tension	4	4.23 (4.72)	9.18 (7.69)	3.15	.002*	64 (84.1)	24 (80)	40 (86.96)	0.66	.416
Heart rate	3	2.24 (3.36)	7.05 (7.00)	3.50	.001*	55 (72.37)	18 (60)	37 (80.43)	3.79	.052
Respiration	4	3.29 (4.26)	5.30 (5.86)	1.62	.110	49 (64.47)	19 (63.33)	30 (65.22)	0.03	.867
Gastrointestinal	4	2.77 (2.77)	6.07 (6.61)	2.59	.012	59 (77.63)	21 (70)	38 (82.61)	1.66	.197

*M* mean, *SD* standard deviation

*p value < .00625 (Bonferroni correction for multiple comparisons)

## Discussion

This two-part study aimed to explore the applicability of the cognitive model of social anxiety (Clark, 2005; Clark & Wells, 1995) to autistic young people. Part one of the study found that autistic young people had similar perceptions of their own social performance compared to observers overall. Participants who experienced greater social anxiety also received poorer subjective and objective ratings on their social performance. These findings are in contrast to the discrepancy between self and observer ratings that were found in neurotypical children aged 5–11 years with high levels of social anxiety (Cartwright‐Hatton et al., 2003, 2005). They are however more consistent with the findings of a recent experimental study of neurotypical adolescents (Leigh et al., 2021) which reported that self, conversational partner and observer performance ratings were negatively impacted for all participants, i.e. both high and low socially anxious individuals when instructed to engage in self-focused attention and safety behaviours. These contrasting findings might be accounted for by task differences with the filmed speech task used by Cartwright-Hatton et al. (2005) provoking high levels of performance anxiety but not necessarily social anxiety as it was delivered in the presence of a researcher but not peers, did not require interaction and was of relatively short duration (2 min) which may not be long enough for social anxiety processes to influence observable social behaviours, particularly those elicited by social interaction. The conversational/discussion tasks used in the present study and that of Leigh et al. (2021) involved social interaction with peers and were longer, thus potentially allowing social anxiety processes to impact objective social performance. The lack of self-observer discrepancy for those with high social anxiety in the present study also resonates with findings from a systematic review which highlighted that negative self-imagery, associated with SFA, can negatively impact upon *both* self- and observer- ratings of social performance in neurotypical individuals with social anxiety (Ng et al., 2014) Furthermore, the review (Ng et al., 2014) found that self-reported negative self-imagery was more related to how one may be perceived from an observer’s perspective, suggesting that fears of negative evaluation by others during this ongoing self-monitoring processing throughout social interactions, and is associated with heightened social anxiety. Our findings suggest that autistic young people may show similar levels of awareness of their social differences and perceive their social interaction in a similar way as that objectively observed by others and consistent with recent research findings, this is negatively impacted by social anxiety. However, it was unclear from study part one what factors may be significant in contributing towards how autistic young people construe the perception of their social performance, whether it may be related to interoceptive sensibility of bodily sensations related to anxiety, or whether it may be related to cognitive factors such as assessing how one may be perceived from an observer perspective and fear of negative evaluation by others.

Part two of the study found that although social anxiety amongst autistic young people was associated with greater state and trait SFA as well as increased interoceptive sensibility, only interoceptive sensibility or awareness of bodily sensations fully mediated the relationship between subjective social performance rating and social anxiety. Furthermore, those who were more socially anxious reported greater sensibility in bodily sensations that are related to anxiety such as increase in bodily temperature, heart rate and muscle tension, suggesting that there may be some specific physiological markers perceived by the self to be associated with social anxiety. This is in keeping with findings reported by Palser et al. (2018) who note that increased interoceptive sensibility in childhood is associated with anxiety, not autism. Therefore, our findings derived from the use of a state/situational measure of interoceptive sensibility suggest that it may be the somatic sensations associated with anxiety that influences a more negative self-image and greater social anxiety in autistic young people albeit situation specific, though caution should be drawn when inferring direction of causation based on the cross-sectional nature of the study. While self-focused attentional processes at a global level have been consistently reported to play a significant role in the cause and maintenance of social anxiety in the literature (see Norton and Abbott (2016) for a review), there may be complexities in relation to autistic young people which are relevant to our findings. The evidence for the role of self-focused attention in social anxiety is primarily derived from studies of typically developing adults. Enhanced processing of self-relevant information and negative interpretations and memory biases have been identified as critical to the generation and maintenance of social anxiety. However the field is complex and debate remains as to whether enhanced vigilance to external cues is also relevant (Rapee & Heimberg, 1997). Cognitive difference in autism may alter the emphasis in respect of the content and mode of enhanced processing relevant to social anxiety in autism. For example, theory of mind differences relevant to self and others’ mental states may reduce the tendency to focus on self-focused internal cognitions and impaired interoceptive sensibility may mean that internal bodily sensations are more salient and distressing.

Relating back to the cognitive model of social anxiety, negative self-imagery is seen both as a predisposing and maintaining factor for social anxiety (Clark & Wells, 1995). One study that examined the nature of the negative self-imagery in neurotypical adults with social anxiety disorder found that participants reported recurrent images of visceral and visual sensations, which also correlated with recalling memories of aversive interpersonal experiences during early childhood, that may have either led to the development of or exacerbated symptoms of social anxiety in later years (Hackmann et al., 2000). Although we did not ask participants to report any social adversity they may have experienced throughout development, our findings partially resonate with that of Hackmann et al. (2000) by showing that autistic young people who have more negative self-imagery also report more visceral sensations, and enhanced interoceptive sensibility for certain bodily sensations related to autonomic activation during social situations is associated with experiences of elevated social anxiety. Our findings resonate with Pickard et al. (2020) who found that interoceptive sensibility in light of enhanced SFA was associated with increased social anxiety amongst autistic young people.

In Hackmann et al. (2000)’s study, participants reported more visible visceral sensations such as blushing, feeling shaky or appearing physically smaller and appearing less attractive to others, which may be directly associated with their concerns about how they appear in social situations and fears of negative evaluation by others. Although a direct comparison cannot be drawn between the current study and that of Hackmann et al. (2000)’s as we did not explicitly ask autistic young people to report on more visible visceral sensations, autistic young people with greater social anxiety in the current study did report enhanced internal bodily sensations that may not be visible to observers (such as heart rate and muscle tension). It is unclear whether the elevated internal somatic sensations of autistic participants also directly influences their cognitive appraisal of how they may be perceived by others, and future studies may explore to what extent self-reports of elevated internal somatic sensations may correlate with more visible visceral sensations among autistic young people who experience anxiety, to draw direct comparisons between results from the current study and that of Hackmann et al. (2000). One hypothesis is that the enhanced bodily sensations may relate to the developmental pathway model of social anxiety in autism, where greater physiological arousal and difficulties with regulation take more of a driving seat in the development of social anxiety for autistic young people following negative social encounters (Bellini, 2004).

### Clinical Implications

Firstly, our findings suggest that negative performance beliefs are an important element in social anxiety experienced by autistic young people. Our findings also suggest that not only does increased interoceptive sensibility play an important role in mediating the relationship between one’s perceived self-image and social anxiety, but there appears to be specific physiological markers (such as heart rate and muscle tension) that are particularly elevated for those who experience high levels of social anxiety. Although it has been proposed that it may be ITPE (discrepancy between interoceptive sensibility and accuracy) that predicts anxiety in autism rather than interoceptive sensibility per se (Garfinkel et al., 2015, 2016), more recent studies have found that it was interoceptive sensibility and not accuracy that was significantly associated with social anxiety in autistic young people (Palser et al., 2018; Pickard et al., 2020). One clinical implication may be to enhance the psychoeducation component of cognitive behavioural treatments for social anxiety. This would include accessible information about the role physiological symptoms play in anxiety and strategies to reduce the impact of increased physiological arousal during social situations. These treatment elements could precede or be delivered alongside other important ingredients of effective psychological interventions for social anxiety.

Progressive muscle relaxation (PMR) was first proposed by Jacobson (1987) and involves actively teaching clients to distinguish between tension (muscle contraction) and relaxation (muscle release) across specific muscle groups activated in a specific order. PMR has been evaluated for use with individuals who have anxiety disorders such as panic disorder and generalised anxiety disorder as a form of relaxation to alleviate anxiety (Conrad & Roth, 2007; McCallie et al., 2006). For autistic young people, PMR may be potentially beneficial in two ways. First, it may enable them to learn a new coping mechanism to actively manage the somatic sensations associated with anxiety. Second, by actively engaging in muscle contraction before release muscle tension, it may help reduce the potential prediction error between interoceptive accuracy and sensibility. Future research may explore the feasibility and usefulness of PMR for autistic young people as part of treatment for social anxiety.

## Limitations and Future Directions

A number of limitations should be taken into consideration when interpreting findings from the current study. First, the sample size for this study was relatively small and therefore we were unable to explore whether there may be sex differences in how interoceptive sensibility and SFA may have influenced both self-performance ratings and social anxiety in autistic youth. The samples only included autistic individuals without co-occurring intellectual disability, and future studies should explore the generalisability of current findings in autistic individuals who may be minimally verbal and/or have cognitive impairment. Participants were also predominantly recruited within settings where small groups may have already formed, potentially increasing familiarity among group participants, reducing anxiety in the social situation and result in underestimation of social anxiety that participants may experience in more unfamiliar settings. Therefore, future studies may seek to recruit a larger group of participants and control for degree of familiarity between group members when assessing social anxiety.

The lack of measurement of autism symptom severity is also a limitation. We were not able to evaluate the contribution, if any, of social communication difference to performance ratings. Furthermore, future studies may explore whether the mediating role played by interoceptive sensibility may stand when controlling for individual differences in autism symptom severity. We also did not include a measure of interoceptive accuracy to help us assess potential ITPE differences for participants with high versus low social anxiety. Future studies should directly compare to what extent interoceptive sensibility, accuracy, and ITPE may account for the relationship between self-imagery and social anxiety for autistic young people, as well as explore whether ITPE may be consistent across different physiological markers which include but is not exclusive to heart rate. Measures of interoceptive sensibility should ideally be contemporaneous and situation-specific to truly understand the relationship between different indices of interoceptive processing. Understanding specific physiological markers where greater ITPE may be observed can help identify targets for clinical intervention which address management of physiological arousal amongst autistic young people who experience high social anxiety.

## Figures and Tables

**Fig. 2:**
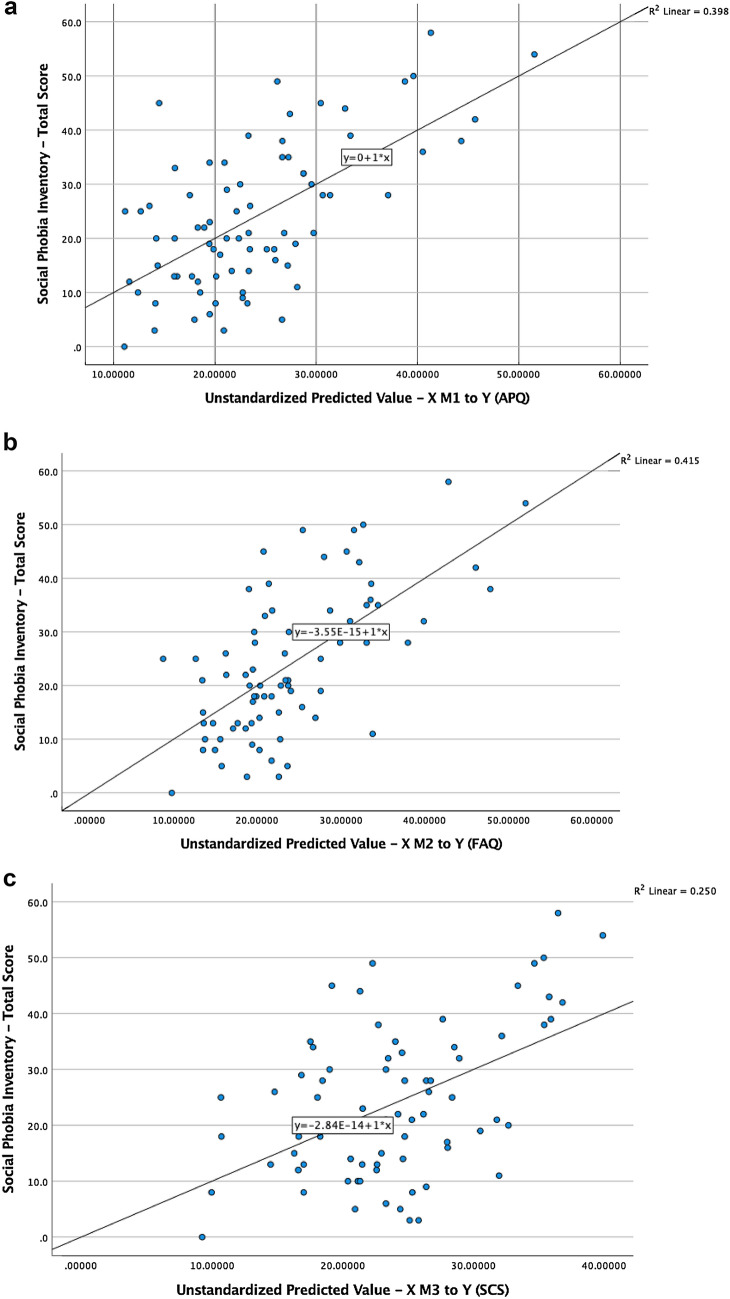
Scatterplots showing social anxiety scores (as measured by social phobia inventory; SPIN) as predicted by self-performance rating (SPR) and **a** interoceptive sensibility (measured by Autonomic Perception Questionnaire; APQ), **b** state self-focused attention (measured by Focus of Attention Questionnaire; FAQ); **c** trait self-focused attention (measured by Social Consciousness Questionnaire; SCS)

## Measures

*Social Anxiety Scale for Adolescents* (SAS-A, La Greca & Lopez, 1998). The SAS-A is an 18-item questionnaire assessing social anxiety in adolescents. Questions are rated using a 5-point Likert scale (1 = ‘not at all’, 4 = ‘all the time’). Higher total scores (range: 18–90) indicate higher social anxiety. SAS-A has excellent internal consistency (Cronbach’s α = 0.92) and modest test–retest reliability (*r* = 0.60) (Storch et al., 2004). The SAS-A has been used with autistic adolescents (Lei et al., 2019, 2020), and internal consistency in the current study was good (Cronbach’s α = 0.88).

*Social Phobia Inventory* (SPIN, Connor et al., 2000) The SPIN is a 17-item questionnaire assessing social anxiety with three sub-scales: fear, avoidance and physiological sensations. Questions are rated using a 5-point Likert scale (0 = ‘not at all’, 4 = ‘extremely’). Higher total scores (range: 0–68) indicate higher social anxiety. The SPIN has good internal consistency (Cronbach’s α = 0.92) and test–retest reliability (r = 0.86) in typically developing adolescents, as well as good convergent validity with the Social Anxiety Scale for Adolescents (r = 0.82) (Johnson et al., 2006). Internal consistency in the current study was excellent (Cronbach’s α = 0.92).

*Focus of Attention Questionnaire* (FAQ, Woody, 1996). A modified version of the FAQ as described in Mellings and Alden (2000) was used to assess SFA during the social situation. The modified version is a 15-item questionnaire comprising the original 10-items plus five items specific to social anxiety, and assesses two domains: SFA (e.g., ‘I was focusing on what I would say or do next’) and other-focused attention (e.g., ‘I was focusing on what the other people were saying or doing’). Questions are rated using a 5-point Likert scale (1 = ‘not at all’; 5 = ‘very much’), with average scores computed for each domain. The original FAQ has excellent internal consistency in typically developing adults (Woody, 1996), whilst Cronbach’s alpha of the modified version was 0.87 for the SFA scale and 0.49 for the OFA scale (Mellings & Alden, 2000). The original FAQ has been used in typically developing adolescents (e.g., Higa & Daleiden, 2008), but neither version has been used with autistic individuals. Internal consistency in the current study was good (Cronbach’s α = 0.82).

*Autonomic Perception Questionnaire* (APQ, Mandler et al., 1958). A modified version of the specific form of APQ was used to assess the interoceptive sensibility during the social situation. The modified APQ contains 23 items assessing the presence of physiological sensations within seven domains: general sensibility, blood pressure, temperature, perspiration, muscle tension, heart rate, respiration, and gastrointestinal symptoms. Items are rated by marking a line representing a 10-point Likert scale (0 = ‘never’, 9 = ‘always’). Higher total scores (range: 0–207) indicate greater interoceptive sensibility. Modifications for use with autistic individuals included: (1) simplifying language to concrete rather than metaphorical terminology, (2) organising items in to ‘body area’ groups (e.g., hands, stomach), (3) adding images to aid understanding, (4) adding extreme and mid-point numbers to the line, (5) grading the colour of the scale from blue to red to represent intensity of feeling, and (6) ensuring left to right ordering of the Likert scale. Internal consistency in the current study was excellent (Cronbach’s α = 0.90).

*Performance Scale* (Cartwright‐Hatton et al., 2003, 2005). A modified version of the Performance Scale was used to assess self- and observer-ratings of social performance during the social situation. The original measure comprised of eight questions in relation to micro-behaviours (e.g., how loud and clear was your voice?), nervous behaviours (e.g., How much did you blush?) and global impressions (e.g., how friendly did you look?). Cronbach’s alpha for the original questionnaire was 0.74 for the child (self-rated) version and 0.91 for the observer version. The modification in the current study added an item relating to the use of eye contact. Items were presented as statements rather than questions and participants were asked to indicate how much they agreed with each statement. Items were rated using a 4-point scale from ‘4’–‘strongly agree’ to ‘1’—‘strongly disagree’. Higher total scores (range: 9–36) indicate better performance. The internal consistency for both self- and observer ratings in the current study were good (Cronbach’s α = 0.82, 0.87 respectively).

*Self-Consciousness Scale* (SCS, Fenigstein et al., 1975). The SCS, a measure of trait SFA, is a 21-item questionnaire with three sub-scales: public self-consciousness (attention to aspects of the self-observable to others), private self-consciousness (internal self-reflection) and social anxiety. Items are rated using a 5-point Likert scale (0 = ‘extremely uncharacteristic’, 4 = ‘extremely characteristic’), with mean scores computed for each subscale and higher scores indicating greater self-consciousness. The SCS has been used in typically developing adolescents (e.g., Davis & Franzoi, 1991; Franzoi & Davis, 1991), and autistic adults (e.g., Blackshaw et al., 2016; Lombardo et al., 2007).

The SCS and APQ were chosen as these were well established measures of the constructs of interest in the present study. There has been some use of the SCS previously in studies of autistic adults (e.g., Lombardo et al., 2007). The APQ is also the measure with most face validity in respect of asking participants to rate experience of bodily sensations although we modified the presentation and format of the measure to make it more accessible for autistic adolescents. The APQ items were modified by including a visual cue at the start of a group of items to illustrate the part of the body being enquired about and specifying that each item referred to the individual’s experience during the group discussion task. Terms were amended by adding concrete detail to explain the descriptors of bodily sensations. For example, in the APQ, individuals are asked to rate changes in the intensity of their heartbeat by indicating on a line as below:

We amended this item by enquiring:

Where terminology might not be accessible, we rephrased some items accordingly. For example, we amended:

to:

For the SPIN, we required a measure of social anxiety that could be administered across a broad age range. While the SPIN was primarily developed for adults 18 years and over, it has also demonstrated acceptable reliability for the measurement of social phobia in adolescents (Ranta et al., 2007). (See Fig. 2).

## Footnotes

1 This paper will use identity-first language, in line with preferences shown by autistic individuals as shown by Kenny et al. (2016).

## References

[CR1] American Psychiatric Association (2013). Diagnostic and statistical manual of mental disorders (DSM-5®).

[CR2] Anderson ER, Hope DA (2009). The relationship among social phobia, objective and perceived physiological reactivity, and anxiety sensitivity in an adolescent population. Journal of Anxiety Disorders.

[CR3] Arain M, Haque M, Johal L, Mathur P, Nel W, Rais A, Sandhu R, Sharma S (2013). Maturation of the adolescent brain. Neuropsychiatric Disease and Treatment.

[CR4] Arora I, Bellato A, Ropar D, Hollis C, Groom MJ (2021). Is autonomic function during resting-state atypical in autism: A systematic review of evidence. Neuroscience & Biobehavioral Reviews.

[CR5] Bellini S (2004). Social skill deficits and anxiety in high-functioning adolescents with autism spectrum disorders. Focus on Autism and Other Developmental Disabilities.

[CR6] Blackshaw AJ, Kinderman P, Hare DJ, Hatton C (2016). Theory of mind, causal attribution and paranoia in asperger syndrome. Autism.

[CR7] Cartwright-Hatton S, Hodges L, Porter J (2003). Social anxiety in childhood: The relationship with self and observer rated social skills. Journal of Child Psychology and Psychiatry.

[CR8] Cartwright-Hatton S, McNally D, White C, Verduyn C (2005). Parenting skills training: An effective intervention for internalizing symptoms in younger children?. Journal of Child and Adolescent Psychiatric Nursing: Official Publication of the Association of Child and Adolescent Psychiatric Nurses, Inc,.

[CR10] Clark DM, Crozier R, Alden LE (2001). A cognitive perspective on social phobia. International handbook for social anxiety.

[CR11] Clark DM, Crozier R, Alden LE (2005). Chapter 9: A cognitive perspective on social phobia. The essential handbook of social anxiety for clinicians.

[CR12] Clark DM, Wells A, Heimberg RG, Liebowitz MR, Hope DA, Schneier FR (1995). A cognitive model of social phobia. Social phobia: Diagnosis, assessment, and treatment.

[CR13] Connor KM, Davidson JRT, Churchill LE, Sherwood A, Weisler RH, Foa E (2000). Psychometric properties of the social phobia inventory (SPIN): New self-rating scale. The British Journal of Psychiatry.

[CR14] Conrad A, Roth WT (2007). Muscle relaxation therapy for anxiety disorders: It works but how?. Journal of Anxiety Disorders.

[CR15] Corbett BA, Muscatello RA, Kim A, Patel K, Vandekar S (2021). Developmental effects in physiological stress in early adolescents with and without autism spectrum disorder. Psychoneuroendocrinology.

[CR16] Craig A (2003). Interoception: The sense of the physiological condition of the body. Current Opinion in Neurobiology.

[CR17] Davis MH, Franzoi SL (1991). Stability and change in adolescent self-consciousness and empathy. Journal of Research in Personality.

[CR18] Domschke K, Stevens S, Pfleiderer B, Gerlach AL (2010). Interoceptive sensitivity in anxiety and anxiety disorders: An overview and integration of neurobiological findings. Clinical Psychology Review.

[CR19] Fenigstein A, Scheier MF, Buss AH (1975). Public and private self-consciousness: Assessment and theory. Journal of Consulting and Clinical Psychology.

[CR20] Franzoi SL, Davis MHA (1991). Stability and change in adolescent self-consciousness and empathy. Journal of Research in Personality.

[CR21] Garfinkel SN, Seth AK, Barrett AB, Suzuki K, Critchley HD (2015). Knowing your own heart: Distinguishing interoceptive accuracy from interoceptive awareness. Biological Psychology.

[CR22] Garfinkel SN, Tiley C, O’Keeffe S, Harrison NA, Seth AK, Critchley HD (2016). Discrepancies between dimensions of interoception in autism: Implications for emotion and anxiety. Biological Psychology.

[CR23] Hackmann A, Clark DM, McManus F (2000). Recurrent images and early memories in social phobia. Behaviour Research and Therapy.

[CR24] Hayes AF (2017). Introduction to mediation, moderation, and conditional process analysis: A regression based approach.

[CR25] Higa CK, Daleiden EL (2008). Social anxiety and cognitive biases in non-referred children: The interaction of self-focused attention and threat interpretation biases. Journal of Anxiety Disorders.

[CR26] Hill EL (2017). IBM SPSS statistics [Windows].

[CR27] Hope DA, Heimberg RG, Klein JF (1990). Social anxiety and the recall of interpersonal information. Journal of Cognitive Psychotherapy.

[CR28] Ingram RE (1990). Self-focused attention in clinical disorders: Review and a conceptual model. Psychological Bulletin.

[CR29] Jacobson E (1987). Progressive relaxation. The American Journal of Psychology.

[CR30] Jefferies P, Ungar M (2020). Social anxiety in young people: A prevalence study in seven countries. PLoS ONE.

[CR31] Johnson HS, Inderbitzen-Nolan HM, Anderson ER (2006). The social phobia inventory: Validity and reliability in an adolescent community sample. Psychological Assessment.

[CR32] Kane L, Ashbaugh AR (2017). Simple and parallel mediation: A tutorial exploring anxiety sensitivity, sensation seeking, and gender. Tutorials in Quantitative Methods for Psychology.

[CR33] Kenny L, Hattersley C, Molins B, Buckley C, Povey C, Pellicano E (2016). Which terms should be used to describe autism? Perspectives from the UK autism community. Autism.

[CR34] Kuusikko S, Pollock-Wurman R, Jussila K, Carter AS, Mattila M-L, Ebeling H, Pauls DL, Moilanen I (2008). Social anxiety in high-functioning children and adolescents with autism and asperger syndrome. Journal of Autism and Developmental Disorders.

[CR100] La Greca, A. M. (1999). *The Social Anxiety Scales for Children and Adolescents*. The Behavior Therapist.

[CR35] La Greca AM, Lopez N (1998). Social anxiety among adolescents: Linkages with peer relations and friendships. Journal of Abnormal Child Psychology.

[CR36] Lebel C, Deoni S (2018). The development of brain white matter microstructure. NeuroImage.

[CR37] Lei J, Ashwin C, Brosnan M, Russell A (2019). Differences in anxieties and social networks in a group-matched sample of autistic and typically developing students transitioning to university. Autism.

[CR38] Lei J, Brosnan M, Ashwin C, Russell A (2020). Evaluating the role of autistic traits, social anxiety, and social network changes during transition to first year of university in typically developing students and students on the autism spectrum. Journal of Autism and Developmental Disorders.

[CR39] Lei J, Russell A (2020). I have a fear of negative evaluation, get me out of here! examining latent constructs of social anxiety and autistic traits in neurotypical and autistic young people. Journal of Autism and Developmental Disorders.

[CR40] Leigh E, Chiu K, Clark DM (2021). Self-focused attention and safety behaviours maintain social anxiety in adolescents: An experimental study. PLoS ONE.

[CR41] Lombardo MV, Barnes JL, Wheelwright SJ, Baron-Cohen S (2007). Self-referential cognition and empathy in autism. PLoS ONE.

[CR42] Makkar SR, Grisham JR (2011). Social anxiety and the effects of negative self-imagery on emotion, cognition, and post-event processing. Behaviour Research and Therapy.

[CR43] Mandler G, Mandler JM, Uviller ET (1958). Autonomic feedback: The perception of autonomic activity. Journal of Abnormal Psychology.

[CR44] Mansell W, Clark DM (1999). How do I appear to others? Social anxiety and processing of the observable self. Behaviour Research and Therapy.

[CR45] Mason J, Scior K (2004). ‘Diagnostic overshadowing’ amongst clinicians working with people with intellectual disabilities in the UK. Journal of Applied Research in Intellectual Disabilities.

[CR46] Mauss I, Wilhelm F, Gross J (2004). Is there less to social anxiety than meets the eye? Emotion experience, expression, and bodily responding. Cognition and Emotion.

[CR47] McCallie MS, Blum M, Hood CJ (2006). Progressive muscle relaxation. Journal of Human Behavior in the Social Environment.

[CR9] Meet the Superhumans. (2012). [Trailer]. Tom Tagholm. dir. London: 4Creative.

[CR48] Mellings TM, Alden LE (2000). Cognitive processes in social anxiety: The effects of self-focus, rumination and anticipatory processing. Behaviour Research and Therapy.

[CR49] National Institute for Health and Care Excellence. (2013). *Social anxiety disorder: Recognition, assessment and treatment*. Clinical Guideline. https://www.nice.org.uk/guidance/cg159. 31869048

[CR50] Ng AS, Abbott MJ, Hunt C (2014). The effect of self-imagery on symptoms and processes in social anxiety: A systematic review. Clinical Psychology Review.

[CR51] Norton AR, Abbott MJ (2016). Self-focused cognition in social anxiety: A review of the theoretical and empirical literature. Behaviour Change.

[CR52] Palser ER, Fotopoulou A, Pellicano E, Kilner JM (2018). The link between interoceptive processing and anxiety in children diagnosed with autism spectrum disorder: Extending adult findings into a developmental sample. Biological Psychology.

[CR53] Pickard H, Hirsch C, Simonoff E, Happé F (2020). Exploring the cognitive, emotional and sensory correlates of social anxiety in autistic and neurotypical adolescents. Journal of Child Psychology and Psychiatry.

[CR54] Pozo C, Carver CS, Weflens AR, Scheier MF (2016). Social anxiety and social perception: Construing others’ reactions to the self. Personality and Social Psychology Bulletin.

[CR55] Ranta K, Kaltiala-Heino R, Koivisto A-M, Tuomisto MT, Pelkonen M, Marttunen M (2007). Age and gender differences in social anxiety symptoms during adolescence: The social phobia inventory (SPIN) as a measure. Psychiatry Research.

[CR56] Rapee RM, Heimberg RG (1997). A cognitive-behavioral model of anxiety in social phobia. Behaviour Research and Therapy.

[CR57] Simonoff E, Pickles A, Charman T, Chandler S, Loucas T, Baird G (2008). Psychiatric disorders in children with autism spectrum disorders: Prevalence, comorbidity, and associated factors in a population-derived sample. Journal of the American Academy of Child & Adolescent Psychiatry.

[CR58] Spain D, Sin J, Linder KB, McMahon J, Happé F (2018). Social anxiety in autism spectrum disorder: A systematic review. Research in Autism Spectrum Disorders.

[CR59] Storch EA, Masia-Warner C, Dent HC, Roberti JW, Fisher PH (2004). Psychometric evaluation of the social anxiety scale for adolescents and the social phobia and anxiety inventory for children: construct validity and normative data. Journal of Anxiety Disorders.

[CR60] Vassilopoulos S (2005). Social anxiety and the effects of engaging in mental imagery1. Cognitive Therapy and Research.

[CR61] White SW, Bray BC, Ollendick TH (2012). Examining shared and unique aspects of social anxiety disorder and autism spectrum disorder using factor analysis. Journal of Autism and Developmental Disorders.

[CR62] Wood JJ, Gadow KD (2010). Exploring the nature and function of anxiety in youth with autism spectrum disorders. Clinical Psychology: Science and Practice.

[CR63] Woody SR (1996). Effects of focus of attention on anxiety levels and social performance of individuals with social phobia. Journal of Abnormal Psychology.

